# Facile Synthesis of Carbon Nanospheres with High Capability to Inhale Selenium Powder for Electrochemical Energy Storage

**DOI:** 10.3390/ma14226760

**Published:** 2021-11-10

**Authors:** Mustafa Khan, Xuli Ding, Hongda Zhao, Xinrong Ma, Yuxin Wang

**Affiliations:** 1 School of Materials Science and Engineering, Jiangsu University of Science and Technology, Zhenjiang 212100, China; mkhan.esd@gmail.com; 2 School of Science, Jiangsu University of Science and Technology, Zhenjiang 212100, China; zhd787278660@163.com (H.Z.); maxinrong2021@163.com (X.M.)

**Keywords:** carbon nanospheres, selenium, Li–Se battery

## Abstract

Carbon–selenium composite positive electrode (CSs@Se) is engineered in this project using a melt diffusion approach with glucose as a precursor, and it demonstrates good electrochemical performance for lithium–selenium batteries. X-ray diffraction (XRD) and scanning electron microscopy (SEM) with EDS analysis are used to characterize the newly designed CSs@Se electrode. To complete the evaluation, electrochemical characterization such as charge–discharge (rate performance and cycle stability), cyclic voltammetry (CV), and electrochemical impedance spectroscopy (EIS) tests are done. The findings show that selenium particles are distributed uniformly in mono-sized carbon spheres with enormous surface areas. Furthermore, the charge–discharge test demonstrates that the CSs@Se cathode has a rate performance of 104 mA h g^−1^ even at current density of 2500 mA g^−1^ and can sustain stable cycling for 70 cycles with a specific capacity of 270 mA h g^−1^ at current density of 25 mA g^−1^. The homogeneous diffusion of selenium particles in the produced spheres is credited with an improved electrochemical performance.

## 1. Introduction

Due to the increasing environmental damage caused by the massive burning of coal, gasoline, and natural gas, there is a growing demand for the effective use of clean energy. Storage of energy is critical for a clean energy system. The ever-increasing need for energy storage can be met by rechargeable batteries with high specific capacity and energy density [1,2,3,4,5]. Due to their high specific energy density and specific capacity, lithium–sulfur batteries are popular among diverse battery systems [6,7]. However, the weak conductivity of sulfur, the considerable volume change during lithium-ion insertion and extraction, and the dissolution of intermediary polysulfides limit their practical application. As a result, much effort has been expended in the hunt for novel cathode materials for rechargeable lithium batteries [8].

Selenium, which has electrochemical characteristics comparable to sulfur, can produce selenides when it reacts with lithium. Selenium has a theoretical gravimetric capacity of roughly 670 mA h g^−1^, which is lower than sulfur (S), but its higher density compensates for this shortcoming. Furthermore, selenium has a higher electrical conductivity (1 × 10^−3^ S m^−1^) than sulfur (5 × 10^−28^ S m^−1^) [9,10] resulting in good rate and cyclic performance without the requirement for a large amount of conductive carbon. Due to these properties, selenium is a good contender for high energy positive electrode material [11]. However, selenium, like sulfur, is susceptible to the dissolution of higher order polyselenides, resulting in rapid capacity degradation, low coulombic efficiency, and poor cyclic performance. Several experiments have been proposed by researchers in recent years to solve these challenges; among these tests, creating a carbon–selenium composite cathode proves to be a good strategy [12,13]. These composites not only diminish the shuttle effect induced by unwanted polyselenides, but they also improve electrical conductivity [14,15,16,17,18,19,20,21,22,23,24,25]. Much work has been done in recent years to construct the carbon–selenium from various carbonaceous materials such as CNTs, PAN [14], and others [26]. Wu et al. [12] developed a carbon–selenium composite by low-temperature ball milling of a Se/PAN combination, which demonstrated a discharge capacity of 244 mA h g^−1^ at 100 mA g^−1^ after 10 cycles. Abouimrane et al. [27] created a selenium–carbon nanotube (Se/CNT) composite as a positive electrode material with a reversible capacity of 350 mA h g^−1^ at 50 mA g^−1^ current density.

Despite the fact that the performance of selenium composite cathodes has increased over time, there is still insufficient contact between the active material and the electronic conductors. Many attempts have been made to improve the electrochemical performance of lithium–selenium batteries; for example, a porous carbon structure has proven to be an effective positive electrode. Wang et al. [28] injected selenium into porous carbon to create a carbon–selenium composite that performed well at 600 °C. However, this is a time-consuming and complex technology, and the present requirement is to design a simple yet effective synthesis procedure. Carbon spheres (CSs) are produced in this contribution using a simple, one-step hydrothermal technique. The carbon–selenium composite improves electrochemical performance when used as a positive electrode. The favorable profiles are due to the incorporation of selenium powder into the CSs, which results in the reduced development of the intermediary polyselenides, which normally dissolve in the electrolytes and cause capacity fading.

## 2. Experimental Section

Synthesis of CSs@Se: All the chemical reagents used in this experiment were purchased from Sigma Aldrich and were not purified further. Carbon spheres (CSs) were created using a straightforward one-step hydrothermal procedure. A known amount of glucose (11.2 g) was introduced to 130 mL of de-ionized water and swirled for half an hour with a constant magnetic stirrer to achieve uniform dispersion. The solution was then poured to two Teflon-lined autoclaves (100 mL each), and the reaction was carried out in a vacuum oven at 180 °C for 6 h to obtain a regular and uniform shape. The autoclaves were then cooled naturally in air to room temperature, and the black solid precipitates were recovered by centrifuge after three washes with ethanol and water (three times each) and desiccated at 80 °C for 12 h (named CSs) in a vacuum oven. To produce CSs@Se, known quantities of carbon spheres and selenium powder (1:2 mass ratio) were mashed together. The mixture was then transferred to a tubular furnace (OTF-1200X) and heated at a rate of 5 °C min^−1^ up to 400 °C, remaining for 3 h in an Ar/H_2_ protection gas. Following that, a sample of CSs@Se was obtained through natural cooling. The synthesized materials were characterized using different microstructural techniques and electrochemical tests for Li–Se batteries.

## 3. Material Characterization

Using a field-emission scanning electron microscope (FESEM, JEOL JMS-7001-F, JEOL, Tokyo, Japan), we determined the structure and morphology of CSs and CSs@Se composite. The EDS instrument installed in the SEM was used to determine the elemental mapping of the elements. The phase composition of the material was determined using an X’Pert PRO diffractometer (XRD, Shimadzu, Kyoto, Japan: XRD-6000, Cu-Kα radiation, 0.15406 nm, λ = 1.5406 Å), with the measuring angle ranging between 5–70° and a scanning rate of 0.8°/s. The thermogravimetry (TG) examination was carried out using a vertical zero friction dilatometer L75VS Linseis (Selb, Germany) at temperatures ranging from 30 to 800 °C in an argon environment in order to determine the selenium weight percent contained in the composite material, and the surface area of the material was calculated using the Brunauer–Emmett–Teller (BET) (ASAP2020) technique.

## 4. Electrochemical Characterization

Using a half-cell, the electrochemical performance of bare carbon spheres (CSs) and CSs@Se composite was investigated. The cathodes were made by combining 70 weight percent active material (CSs/CSs@Se), 10 weight percent PVDF binder, and 20 weight percent carbon black (Super-P). A small amount of N-methyl pyrrolidone (NMP) was also added to the mixture, which was then agitated for 12 h with a continual magnetic stirrer to achieve a homogenous mixture. Finally, this homogeneous mixture (slurry) was applied on the foil (copper). The coated foil was then dried overnight at 60 °C. The foil was punched into disc shapes to make electrodes. The anode was a lithium foil disc with a 1M LiPF_6_ solution in an electrolyte mixture of Ethyl carbonate (EC) and Diethyl carbonate (DEC) (1:1, *v*/*v*) and a porous Celgard 2400 separator. Half-cells were produced in a glove box with argon flowing (H_2_O < 0.1 PPM, O_2_ < 0.1 PPM), then left for 8 h before being tested for electrochemical performance. The LAND battery testing station was used to undertake rate performance and cyclic stability tests in the voltage range of 0.005–3 V (vs. Li/Li^+^) at various current densities. For the first five cycles, a cyclic voltammogram (CV) test was performed on workstation CH1611E with a voltage window of 0.005–3 V at 0.5 mV/s. The coin cell impedance was measured at 10 mV over a frequency range of 0.001–100,000 Hz.

## 5. Results and Discussion

**Figure 1:**
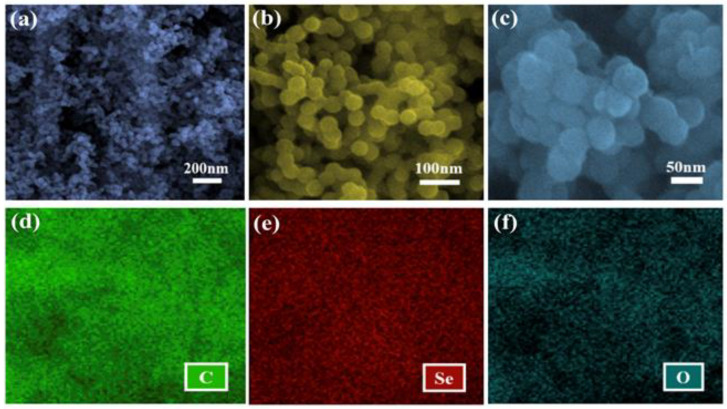
Morphology of bare carbon spheres and CSs@Se composite: (**a**,**b**) low and high magnification SEM images of bare carbon spheres, (**c**) high magnification surface morphology of CSs@Se composite, (**d**–**f**) EDS mapping of the CSs@Se showing the presence of carbon (C), selenium (Se), and oxides (O), respectively.

**Figure 2:**
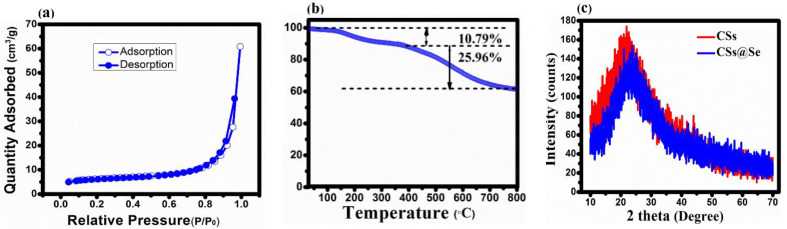
(**a**) Nitrogen adsorption and desorption isotherm of CSs, (**b**) TGA curve of CSs@Se composite, and (**c**) XRD pattern of CSs and CSs@Se composite.

Low and high magnification SEM pictures of bare carbon spheres are shown in Figure 1a,b. Carbon spheres are homogeneous with surface area of 23.59 m^2^/g, as shown in Figure 2a. Figure 1c shows selenium-loaded carbon spheres; loading of the selenium is confirmed by the EDS analysis. The elemental mapping of the CSs@Se composite is shown in Figure 1d–f, indicating that carbon and selenium oxides are also generated during the synthesis process, though in modest amounts.

TGA of CSs@Se composite was conducted in Figure 2b. A weight loss of 10.79 wt% below 350 °C could be attributed to the evaporation of moisture absorbed in pores. From 350 to 800 °C, the weight loss of 25.9 wt% was due to the removal of heteroatoms and the evaporation of selenium contents in the composite. The Gibbs free energy equation is as follows:

(1) $$∆G_{B}=4\pi \sigma \left(r^{2}-\frac{2r^{3}}{3r\prime }\right),$$

where $∆G_{B}$ is Gibbs free energy, r is the radius of carbon spheres, σ is the interfacial tension, and r′ is the critical radius [29]. At the beginning of the hydrothermal process at 180 °C, $∆G_{B}$ > 0 and r’ < r form an embryo, but as time passes, the system’s Gibbs free energy decreases, resulting in the development of carbon nuclei with radius r. X-ray diffraction (XRD) was performed to check if selenium has been successfully loaded in to carbon spheres (Figure 2c). The bare carbon spheres (CSs) show a large diffraction peak at 2 theta = 22.3°; this peak can be indexed to 002 plane, which indicates that CSs are in amorphous phase [30]. After loading of the selenium, no new peak is observed because all selenium is absorbed into the pores. There is small shift in the peak at 2 theta = 23.76°, which are caused by the high temperature and impinging of the selenium into the pores.

**Figure 3:**
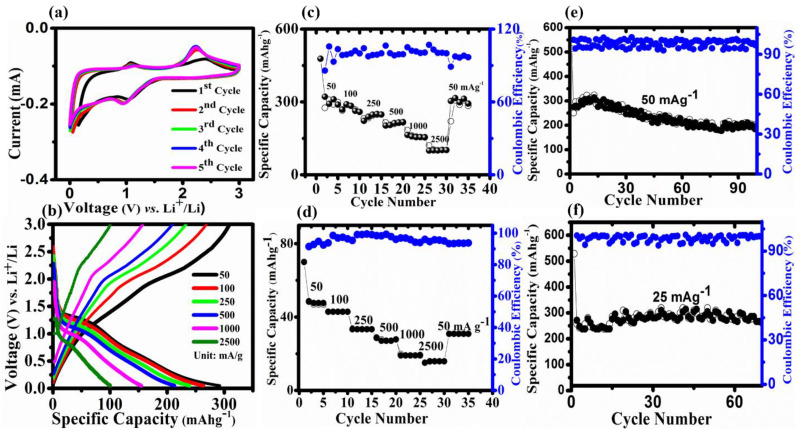
Electrochemical characterization of CSs@Se and CSs: (**a**) cyclic voltammetry (CV) of composite between 0.005-3 V at 0.5 mV s^−1^, (**b**) charging–discharging profiles of CSs@Se at different current rates (50–500 mA g^−1^), (**c**) rate performance of CSs@se (**d**) rate performance of CSs, (**e**) cyclic stability after 100 cycles of CSs@Se at 50 mA g^−1^, and (**f**) cyclic stability after 70 cycles of CSs@Se at current density of 25 mA g^−1^.

The electrochemical testing of a CSs@Se composite positive electrode for lithium–selenium (Li–Se) batteries is performed with a 2032 type coin cell and a lithium foil negative electrode. The cyclic voltammetry (CV) curves of CSs@Se composite at 0.5 mV s^−1^ for five cycles from 0.005–3 V (vs. Li^+^/Li) are shown in Figure 3a. As demonstrated in Figure 3a, the reduction in selenium into insoluble Li_2_Se corresponds to one cathodic peak centered at 1.03 V for the first curve (black one). The occurrence of only one peak indicates that no polyselenides phase exists during the lithiation process and hence no shuttle effect. The presence of two anodic peaks at 1.07 and 2.3 V (for the first curve) suggests that the reduced Li_2_Se was oxidized back during the de-lithiation process. After the first cycle, the anodic and cathodic peaks at 1.07 and 2.6 V have little displacement around 0.96 V for the second cycle, and for the other cycle, the curves practically coincide, indicating that CSs@Se composites are reversible. The charge–discharge pattern at various current values is shown in Figure 3b. The initial discharge curve shows a single slope voltage plateau at 1.03 V, which best matches the CV result and demonstrates single-phase selenium conversion into insoluble Li_2_Se. After the first discharge curve, the slope voltage for the others shifts somewhat. The slope voltage plateaus on the charge curves correlate to the two peaks on the cyclic voltammetry curve.

Meanwhile, Figure 3c,d exhibit the rate performances of CSs@Se composite and CSs at various current densities (50 mA g^−1^ to 2500 mA g^−1^). The composite cathode has an average specific capacity (at current densities) of 294 (50 mA g^−1^), 279 (100 mA g^−1^), 244 (250 mA g^−1^), 209 (500 mA g^−1^), 156 (1000 mA g^−1^), and 104 mA h g^−1^ (2500 mA g^−1^). Furthermore, when the current returns to its initial amount, a specific capacity of 290 mA h g^−1^ is measured, demonstrating that the CSs@Se composite, as a positive electrode, is highly reversible. At the same current densities as above, the capacities of the bare CSs are 47.8, 43.06, 32.6, 26.6, 18.4, and 14.9 mA h g^−1^. Figure 3b depicted the equivalent charge–discharge curves at various current rates. The voltage curve is shown to be reduced at high current values as the current density increases to 2500 mA g^−1^. Due to the limited electrochemical kinetics in the CSs@Se electrode, the Li^+^ are not fully intercalated into the CSs@Se composite at higher densities. Figure 3e,f depict the composite’s cycle performance. The specific capacity of 190 mA h g^−1^ was preserved at 50 mA g^−1^ for 100 cycles, but the specific capacity of 270 mA h g^−1^ was preserved at 25 mA g^−1^.

**Figure 4:**
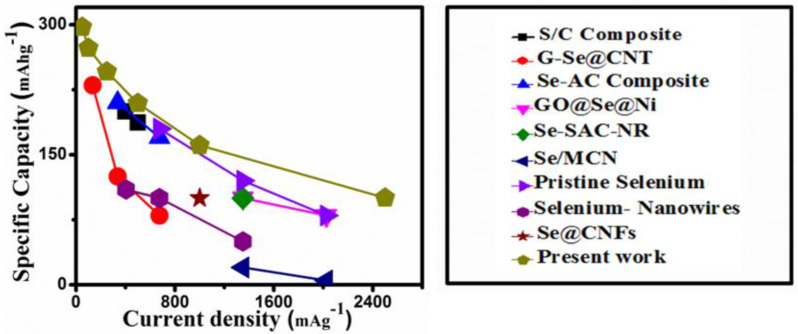
Comparison of current work (rate performance) with that of previously discussed results [15,20,32,33,34,35,36,37] on Li-Se battery.

In addition, the rate performance of the current CSs@Se composite is compared to other materials, such as S/C composite [31], G-Se@CNT [14], Se-AC composite [19], GO@Se@Ni [32], Se-SAC-NR [33], Se/MCN [17], pristine selenium [34], selenium nanowires [35], and Se@CNFs [36]. The comparison in Figure 4 shows that the rate performance of the CSs@Se composite outperforms the previously reported values. This is primarily due to the incorporation of selenium into the carbon spheres, the good consistency of the size of the carbon spheres, the creation of less polyselenide, and the absence of the shuttle effect.

**Figure 5:**
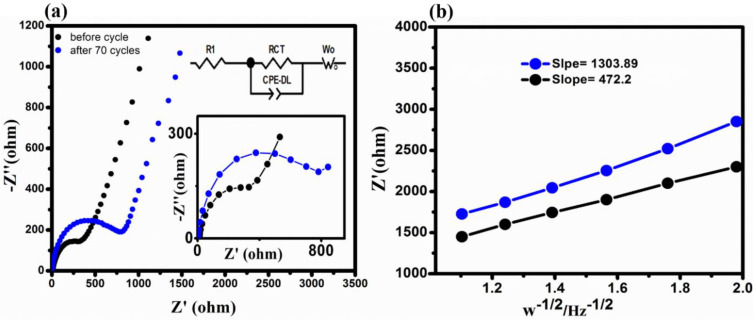
(**a**) The Nyquist plot of CSs@Se composite before and after cycling with Rct values plotted and equivalent circuit inserted. (**b**) Fitted lines of impedance, Z′ vs. angular frequency w^−1/2^ (before cycling and after 70 cycles).

The EIS analysis was then used to look into the charge transferred resistance (Rct) and diffusion coefficient (D) of Li^+^ in the composite. The Rct for CSs@Se composite before and after 70 cycles is shown in Figure 5a. Additionally, according to Z view software analysis, the Rct values for the CSs@Se composite are 338 Ω before cycling, which is significantly lower than the composite after 70 cycles (789 Ω). It shows that, as the number of cycles grows, the diffusion resistance increases, implying that Li ions bear less polarization during intercalation into the CSs@Se composite. Furthermore, Table 1 illustrates the various circuit values of the composite before cycling. Moreover, the diffusion coefficient (D) for Li^+^ can be calculated from the slopes of ω^−1/2^ and Z’ (Figure 5b), where the slope is proportional to the square of the Warburg factor as follows:

(2) $${{D_{\mathrm{Li}}}^{+}}_{\;}\propto \;1/\sigma^{2},$$

This equation is utilized to determine the diffusion coefficient for the composite before and after cycling [37,38,39,40], and the obtained values for the σ for fresh and for the cell after 70 cycles are 472.2 and 1303.89, respectively, as illustrated in Figure 5b.

## 6. Conclusions

A simple hydrothermal procedure was used to successfully create CSs@Se composite, which is used as the positive electrode in lithium–selenium batteries. According to the synthesized substance, it is extremely homogenous and favors easy selenium loading. This composite’s electrochemical performance was evaluated, and it demonstrated good rate performance (294 mA h g^−1^ at 50 mA g^−1^ and 104 mA h g^−1^ even at 2500 mA g^−1^) and cyclic performance (270 mA h g^−1^ even after 100 cycles). The improved electrochemical performance owes to the rapid intercalation/de-intercalation of Li^+^ into the CSs@Se composite and the decreased generation of undesirable soluble-polyselenides.

## Figures and Tables

**Table 1:** Shows the different circuit element values for CSs@Se composite before cycling.

R1	RS	CPE-DL
2.5 Ω	338 Ω	4 × 10^−5^ F

## Data Availability

The data presented in this study are available on request from the corresponding author.
